# Large-scale monitoring of effects of clothianidin dressed oilseed rape seeds on pollinating insects in Northern Germany: implementation of the monitoring project and its representativeness

**DOI:** 10.1007/s10646-016-1724-9

**Published:** 2016-09-27

**Authors:** Fred Heimbach, Anja Russ, Maren Schimmer, Katrin Born

**Affiliations:** 1 tier3 solutions GmbH, Leverkusen, Germany; 2 Spatial Business Integration GmbH, Darmstadt, Germany

**Keywords:** Sublethal effects, Risk assessment, Bee monitoring, Site selection, Spatial analysis, GIS

## Abstract

Monitoring studies at the landscape level are complex, expensive and difficult to conduct. Many aspects have to be considered to avoid confounding effects which is probably the reason why they are not regularly performed in the context of risk assessments of plant protection products to pollinating insects. However, if conducted appropriately their contribution is most valuable. In this paper we identify the requirements of a large-scale monitoring study for the assessment of side-effects of clothianidin seed-treated winter oilseed rape on three species of pollinating insects (*Apis mellifera*, *Bombus terrestris* and *Osmia bicornis*) and present how these requirements were implemented. Two circular study sites were delineated next to each other in northeast Germany and comprised almost 65 km^2^ each. At the reference site, study fields were drilled with clothianidin-free OSR seeds while at the test site the oilseed rape seeds contained a coating with 10 g clothianidin and 2 g beta-cyfluthrin per kg seeds (Elado®). The comparison of environmental conditions at the study sites indicated that they are as similar as possible in terms of climate, soil, land use, history and current practice of agriculture as well as in availability of oilseed rape and non-crop bee forage. Accordingly, local environmental conditions were considered not to have had any confounding effect on the results of the monitoring of the bee species. Furthermore, the study area was found to be representative for other oilseed rape cultivation regions in Europe.

## Introduction

Pollinating insects are a key component of terrestrial ecosystems and provide an essential ecosystem service to wild plants and agricultural crops. The annual value of insect pollination to agriculture was estimated to be worth US$ 153 billion globally in 2005 (Gallai et al. 2009) and the demand of pollination services is high (Aizen and Harder 2009; vanEngelsdorp and Meixner 2010). However, many factors are suspected to impact pollinator health, including parasites, the loss of habitat and decreasing diversity of foraging resources (Goulson et al. 2008; Potts et al. 2010; vanEngelsdorp and Meixner 2010; Winfree et al. 2009). Furthermore, the use of plant protection products (PPPs) has been suggested to harm pollinating insects. In particular the neonicotinoids, a group of systemic insecticides, have been the subject of much discussion about whether they cause adverse effects in pollinating insects under field conditions (e. g., Godfray et al. 2014, Schmuck and Lewis 2016).

Before a new PPP gets authorization for use in Europe, it is subject to an extensive ecotoxicological risk assessment in order to minimize the potential of adverse effects on non-target organisms (European Commission 2009). These risk assessments follow a tiered approach based on worst-case assumptions to ensure cost-effectiveness and proportionality, ranging from laboratory toxicity tests to more complex higher tier studies under field realistic conditions (European Food Safety Authority 2013). The lower tier studies are an important tool to assess intrinsic mechanisms and identify potentially adverse effects of PPP exposure in a set of model organisms. The strength of these studies is their well-defined exposure under controlled laboratory conditions. However, these studies apply artificial conditions regarding the concentration and duration of the exposure to the PPP (Carreck and Ratnieks 2014; Godfray et al. 2014) and simplify or disregard processes which might be of relevance in the field (Cutler and Scott-Dupree 2014; Godfray et al. 2014; Liess et al. 2005). Particular uncertainty exists about the actual exposure of pollinating insects to the focal PPP under field conditions because, for example, bees forage on a wide array of pollen and nectar sources or may actively avoid pollen and nectar from treated crops which might reduce their exposure to the PPP in question. Furthermore, the vulnerability of a species might differ from the model species due to different ecological traits (Decourtye et al. 2013; Liess et al. 2005). Due to these uncertainties, monitoring studies at the landscape level may be needed in addition to the common sequential testing in risk assessment to gain a sound understanding of the actual environmental effects of PPPs under current agricultural practice (Liess et al. 2005).

In the case of clothianidin, a neonicotinoid insecticide which is used in seed dressings of a number of crops e.g. sugar beet, maize and oilseed rape (OSR), concerns were raised by laboratory studies indicating sub-lethal effects in bees (Godfray et al. 2014). However, field studies obtained different results leading to differing conclusions (Blacquière et al. 2012; Cutler et al. 2014; Cutler and Scott-Dupree 2007; Pilling et al. 2013; Pohorecka et al. 2012). In this issue, we present a large-scale monitoring study which examines the potential side effects of clothianidin dressed OSR seeds on bee pollinators under common agricultural practice at the landscape level. This project consisted of four different pollinator studies performed jointly in the project area: a honey bee (*Apis mellifera*) monitoring study (Rolke et al. 2016a), a bumble bee (*Bombus terrestris*) monitoring study (Sterk et al. 2016), a mason bee (*Osmia bicornis*) monitoring study (Peters et al. 2016), and a residue analysis in pollen and nectar collected by the three investigated bee species (Rolke et al. 2016b). Because large-scale monitoring studies with freely foraging bees can be challenging to conduct and interpret due to a range of confounding factors (Godfray et al. 2014), this paper aims to identify the requirements of a large-scale monitoring study and documents how these were implemented in the current study to increase its validity and conclusiveness.

To conduct this large-scale monitoring, the prospective study area was selected based on the following requirements: (i) high density of OSR cultivation with (ii) no other mass flowering crops available as bee forage during OSR flowering, (iii) homogenous environmental conditions over a spatial extent of several thousand hectares which allow delineation of two spatially separate study sites, and (iv) representativeness of the study area for other OSR cultivation sites in a regional and European context.

## Study area and design

**Fig. 1:**
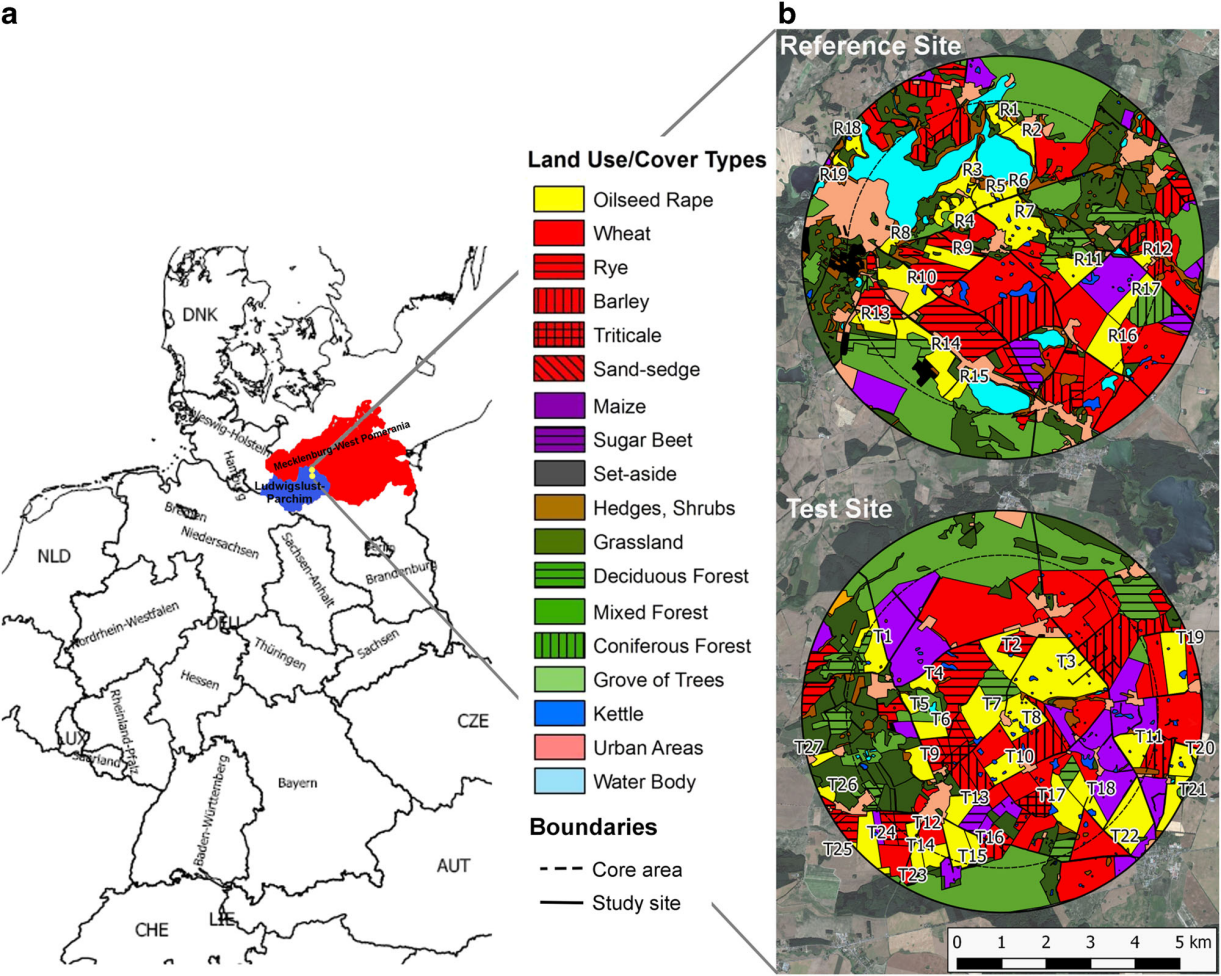
Location of study sites in Central Europe and their habitat composition. **a** The study sites (yellow points) are situated in the district of Ludwigslust-Parchim (blue), which is part of the federal state Mecklenburg-West Pomerania (red). **b** Yellow polygons indicate the numbered OSR fields. The broken inner circle marks the core areas of the study sites of 7 km in diameter

A region in northeast Germany (federal state of Mecklenburg-West Pomerania, Fig. 1a) was identified based on official cropping statistics (Statistical Offices of the Federation and the federal states 2014) and CORINE land cover data (European Environment Agency 2011, Table 1) to meet the requirements of high OSR cultivation density with no other crop providing suitable bee forage during OSR flowering. In the study area, winter OSR is usually cultivated on 25–33 % of the arable land and the agronomic infrastructure of the area with large farms enabled the cooperation with a manageable number of farmers. Two circular study sites of 9 km in diameter were delineated next to each other each containing a core area of 7 km in diameter which were investigated in depth (Fig. 1b). At the reference site (R), study fields were drilled with clothianidin-free OSR seeds while at the test site (T) the OSR seeds contained a coating with 10 g clothianidin and 2 g beta-cyfluthrin per kg seeds (Elado®). Farmers were allowed to decide about all agricultural activities such as sowing, application of fertilisers and PPPs as well as harvesting according to their common practice. This includes—if necessary—the compensation of the missing insecticidal dressing of OSR seeds at the reference site by spray application of insecticides to ensure crop emergence and homogenous flowering. In total, the sites provided nearly 1800 ha of OSR crops (27 % of available arable land) and both study sites covered an area of approximately 65 km^2^ each.

**Table 1:** Overview of (geo)data used for the analyses of the study area and other OSR cultivation regions

Data type	Major data use	Data source
Administrative boundaries	• General study sites identification & characterisation	• NUTS 2 and 3 (Nomenclature des unités territoriales statistiques, Eurostat 2011)
	• Study sites context setting	
Cropping statistics	• General study sites identification & characterisation	• Struktur der Bodennutzung in Mecklenburg-Vorpommern 2014 (Statistisches Amt Mecklenburg-Vorpommern 2015)
	• Study sites context setting	• Regional database Germany (Statistical Offices of the Federation and the federal states 2014)
	• Transfer to EU level	
Land use / land cover (LULC)	• General study sites identification & characterisation	• Corine Land Cover CLC2006 (European Environment Agency 2011)
		• Topographic (arcgis.com)
	• Sites similarity analysis	• Generated high-resolution LULC data by manual digitisation from aerial imagery (www.arcgis.com) and field work
	• Study sites context setting	
	• Transfer to EU level	
Bee forage	• Sites similarity analysis	• Spatially explicit semi-quantitative field sampling (GPS) of bee forage plants and mapping
	• Transfer to EU level	
		• Generated high-resolution LULC data by manual digitisation from aerial imagery (www.arcgis.com) and field work
Weather	• General study sites characterisation	• European Centre for Medium-Range Weather Forecasts (ECMWF) (2000–2013)
	• Sites similarity analysis	
	• Study sites context setting	• German Weather Service (1995–2014)
	• OSR phenology analysis	
Climate	• General study sites characterisation	• European Centre for Medium-Range Weather Forecasts (ECMWF) (2000–2013)
	• Sites similarity analysis	
	• Study sites context setting	• Lauer et al. (2002)
	• Eco-physiological climate classification	
Soil	• Study sites context setting	• Joint Research Centre of the European Community (EC) (1995–2014)
	• Site characterisation and context setting	

The size of the study sites was determined by the number of investigated bee hives and to provide at least 3 km of buffer area around the bee hives to ensure the exclusive exposure to the reference and test conditions, respectively. Although maximum foraging flights of single honey bees have been reported to extend up to 15 km under exceptional conditions (Beekman and Ratnieks 2000), these distances are not representative for whole colonies under attractive foraging conditions (Beekman and Ratnieks 2000; Steffan-Dewenter and Kuhn 2003). Therefore, a minimum of 3 km between the bee hive locations and the border of the study sites was considered adequate to cover the foraging flight distances for honey bees (Eckert 1933; Garbuzov et al. 2015; Steffan-Dewenter and Kuhn 2003) and bumble bees (Darvill et al. 2004; Osborne et al. 2008; Walther-Hellwig and Frankl 2000; Wood et al. 2015). Mason bees on the other hand conduct distinctly shorter foraging flights (Gathmann and Tscharntke 2002) and, hence, their nesting shelters could be positioned closer to the edge of the study site, although the distance always exceeded 1.9 km.

In total, 96 honey and 120 bumble bee hives were positioned at six study locations per study site within the central part of the study sites. Of these study locations, three were situated at the edge of an OSR field and three approximately 400 m apart from the nearest OSR field to allow consideration of suggested impacts on orientation and the homing capability of the bees after exposure to neonicotinoids (Decourtye and Devillers 2010; Henry et al. 2012). The study locations were identical for honey and bumble bees, although their hives were positioned approximately 10–30 m apart from each other. Three nesting shelters with 8 nesting blocks for mason bees were set-up at each of 6 additional study locations per study site (96 nesting shelters in total). As for the other bee species, three of these study locations were situated at the edge of an OSR field and three about 100 m distant from the nearest OSR field, accounting for the shorter foraging flights of the mason bees. According to the number of hives and nesting shelters per study location, there were 8 repetitions for the monitoring of honey bees and mason bees, and 9 repetitions for bumble bees because one of the ten hives per study location was exclusively used for pollen sampling (compare Sterk et al. 2016).

The requirements for separation of reference and test conditions limit the possibility for true statistical replication which would be desirable under ideal conditions (Hurlbert 1984) but is hardly feasible for large-scale, resource intensive studies like large honey bee field trials (European and Mediterranean Plant Protection Organization PP 1/170 (4) 2010; Pilling et al. 2013). Because a possible treatment effect could be confounded with site differences the study sites were carefully chosen so that differences were limited to an absolute minimum achievable under field conditions and the applied mixed effects models are a common tool to address non-independence of data (Zuur et al. 2009). This study design ensured sufficient statistical power to detect even small to medium side effects of clothianidin dressed OSR on the development of hives, reproduction and health of the bees. Further details of the experimental set-up of the bee monitoring studies are given in the respective papers (Peters et al. 2016; Rolke et al. 2016a,b; Sterk et al. 2016).

## Overcoming confounding effects

The main challenge of studies under field realistic conditions is to overcome the diverse confounding factors. Ideally, identical environmental conditions prevail at all investigated study sites to relate any differences found exclusively to the treatment and increase the validity of the results. However, in a large-scale monitoring study like the one presented in this issue, equal conditions cannot be ensured as variability is part of the natural system (Liess et al. 2005). Nevertheless, where variable conditions cannot be avoided, the parameters can be measured and included as covariates in the statistical analyses, thus, providing a better understanding of complex interactions under realistic field conditions.

In the following, the measures applied to account for common uncertainties in field studies are highlighted. These measures include (i) comparable conditions at the reference and test sites in terms of land use, soil, climate, alternative forage resources, as well as development of the OSR, (ii) ensuring the crop fidelity of the studied bees and (iii) the exposure of the bees to the focal neonicotinoid.

### Similarity of environmental conditions and agronomic practice at the reference and test site

#### Land cover and land use types

The study sites were chosen based on the high OSR crop density for the region but also to resemble each other in important environmental conditions. Land use/land cover data (LULC) for the study area were obtained from high-resolution aerial images (Table 1). All types of arable fields as well as different landscape structures such as hedges, kettles (small hollows originating from buried dead ice after glacier retreat), and settlements were identified in the field and manually digitised from satellite images (Google Satellite, date taken 06.05.2011) using a computer-based geographical information system (Quantum GIS, Version 1.8.0 Lisboa). The exact coordinates of the location and shape of OSR fields in the study area as well as relevant landscape features inside the fields such as kettles, forest patches or shrubs were recorded with a GPS handheld receiver (Garmin eTrex 10).

**Fig. 2:**
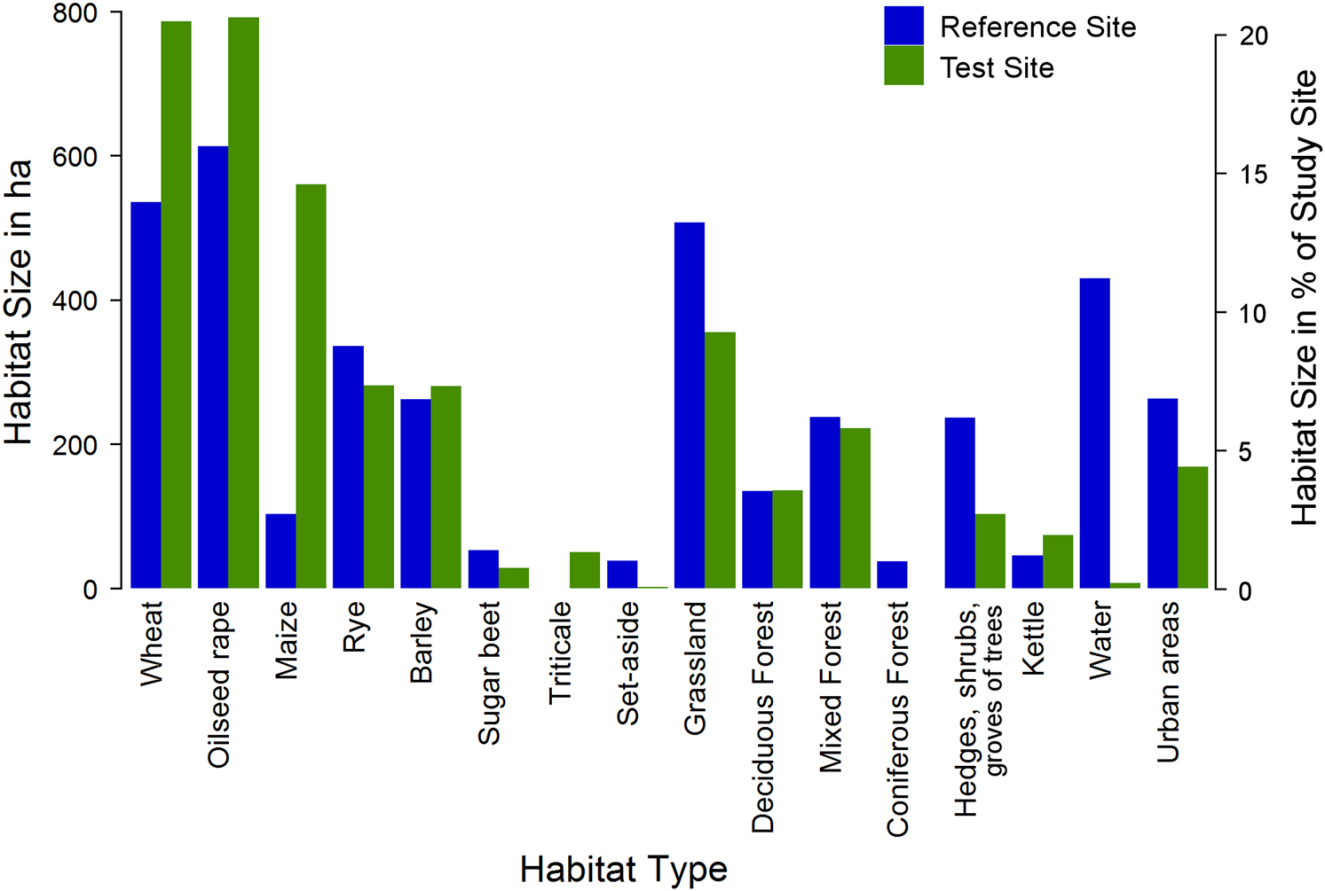
Sizes of habitats and crop types at the reference site (blue) and the test site (green) and their proportion of the core area of the respective study site

The habitat mapping indicated a diverse distribution of different LULC types at both study sites and although field sizes are relatively large (up to several hundreds of ha), the whole area is well structured by a diversity of small forest patches and groves of trees, hedges, water bodies of different sizes and kettles (Fig. 1b). The most important land-use type was arable land, covering 49.5 % and 72.2 % of the core area of the reference site and the test site, respectively. The higher proportion of arable land at the test site was mainly due to the larger cropping area of maize and the lack of any larger water body at the test site (Fig. 1b, Fig. 2). OSR was the most common crop at both study sites (Fig. 2). At the core of the reference site, 17 study fields covered in total 614.6 ha with OSR, constituting 16.0 % of the area, whereas the test site comprised 791.7 ha (20.6 %) of OSR at 18 study fields (Fig. 1b, Fig. 2). The median size of OSR study fields did not differ between reference and test site (Table 2).

**Table 2:** Summary of parameter comparison between the study sites

Dependent variable	Test	Test statistic	*P*	Reference site	Test site
*Land cover*					
Total OSR at Core area				614.6 ha	791.7 ha
Median OSR field size	Wilcoxon rank sum test	W = 170	0.999	33.5 ha	35.3 ha
Percentage OSR of arable land				32.3 %	28.5 %
Percentage arable land				49.5 %	72.2 %
*Soil characterisation of OSR fields*					
pH	Linear mixed model	F_1, 33_ = 3.79	0.060	6.33 ± 0.72	6.15 ± 0.44
Total organic carbon	Linear mixed model	F_1, 33_ = 3.92	0.056	1.02 ± 0.24 %	0.88 ± 0.20 %
Water holding capacity/100 g dry matter	Linear mixed model	F_1, 33_ = 3.71	0.063	28.5 ± 2.0 g	26.9 ± 2.6 g
Soil type	Fisher’s exact test		0.093	98.3 % loamy sand	98.7 % loamy sand
*Climatic conditions at study locations during exposure phase*					
Daily mean temperature	Linear mixed model	F_10, 335_ = 0.19	0.669	12.3 ± 2.4 °C	12.2 ± 2.6 °C
Daily mean of relative humidity	Linear mixed model	F_10, 335_ = 0.50	0.497	77.9 ± 9.3 %	78.5 ± 9.3 %
Daily sum of precipitation	Linear mixed model	F_10, 335_ = 0.12	0.739	1.4 ± 2.2 mm	1.3 ± 3.1 mm
Daily mean wind speed	Linear mixed model	F_10, 308_ = 0.62	0.449	1.4 ± 0.7 m/s	1.5 ± 0.7 m/s
*Agronomic practice*					
Flowering time of OSR varieties by crop area			Early	17.6 %	50.5 %
			Medium	68.6 %	32.1 %
			Late	13.8 %	17.3 %
Thousand seed weight	General linear model	F_1, 32_ = 1.63	0.211	6.86 ± 1.32 g	5.92 ± 1.21 g
Drilling rate	Student’s t-test	t = −3.40	0.002	2.8 ± 0.8 kg/ha	3.6 ± 1.1 kg/ha
Drilling rate weighed by crop area	General linear model	F_1, 65_ = 0.52	0.474	2.6 ± 1.3 kg/ha	2.7 ± 1.7 kg/ha
OSR seeds/m^2^	Wilcoxon rank sum test	W = 304.0	<0.001	39.6 ± 7.4	50.8 ± 14.2
Total number of insecticide spray applications	Wilcoxon rank sum test	W = 192.5	0.021	4.8 ± 0.4	4.1 ± 0.9
Number of insecticide spray applications in autumn 2013	Wilcoxon rank sum test	W = 195.5	0.016	1.4 ± 0.8	0.9 ± 0.25
Number of insecticide spray applications in spring 2014	Wilcoxon rank sum test	W = 152.0	0.543	3.4 ± 0.5	3.1 ± 0.9
*OSR development*					
Date of OSR drilling	ANOVA	F_1, 33_ = 1.09	0.305	18 August 2013 ± 2.5 days	19 August 2013 ± 4.4 days
OSR emergence rate	Wilcoxon rank sum test	W = 669.5	0.800	68 ± 21 %	68 ± 28 %
OSR plant density	Wilcoxon rank sum test	W = 391.5	0.004	26.0 ± 7.3 plants/ m^2^	32.6 ± 11.4 plants/m^2^
OSR development (BBCH stages)	ANOVA	F_1, 232_ = 0.00	0.972		
OSR yield	General linear model	F_1, 33_ = 8.08	0.008	33.9 ± 7.1 dt/ha	38.6 ± 6.3 dt/ha
*Clothianidin*					
Residues in soil before drilling^a^	Linear mixed model	F_1, 33_ = 0.53	0.470	1.9 ± 1.1 μg/kg	2.3 ± 1.5 μg/kg
Loading of OSR seeds	Linear mixed model	F_1, 32_ = 439	<0.001	0.06 ± 0.07 g/kg	7.8 ± 1.5 g/kg
Application rate	Linear mixed model	F_1,32_ = 83.9	<0.001	0.19 ± 0.25 g/ha	28.8 ± 10.0 g/ha

Mean values are given ± standard deviation. For parameters without test statistic, only descriptive analyses were performed

^a^ Calculating with upper limits of 1.5 μg/kg for determined concentrations <LOD_soil_ and 5 μg/kg for <LOQ_soil_

#### Soil characterisation

To characterize the soil from each study field of the core areas, soil samples were taken before drilling of OSR seeds in August 2013. Study fields were subdivided into plots of 10 ha. Ten samples from equally spaced points were taken from the upper 10 cm of the soil in each plot. Plant material and other coarse contaminants were removed and all samples of one plot were combined and thoroughly mixed before the analyses. Characterisation of soil samples included the determination of pH (DIN ISO 10390 (2005)), total organic carbon (TOC, DIN ISO 10694 (1996)), water holding capacity (WHC, DIN EN ISO 11274 (1998)), and particle size (DIN 19683 (2012)). The pH, TOC and WHC were tested for differences between the study sites by fitting linear mixed models which included the study field ID as a random effect to account for the non-independence of sampled plots per study field. The soil type classification was analysed with a Fisher’s exact test. The soil characterisation indicated no significant difference between study fields at the reference and test site regarding the pH, the total organic carbon, and the water holding capacity (Table 2). The soil texture was identified to contain, on average, 67 % sand, 23 % silt, and 10 % clay and was classified accordingly as predominantly loamy sand both at the reference (98.3 %) and test site (98.7 %). Loamy sands are dominated by sand particles, but contain enough clay and silt to provide some structure and fertility.

#### Climatic conditions

To account for small scale climatic differences, weather conditions were measured at all study locations during the exposure phase. At each honey bee location, calibrated devices connected to two hive balances (CAPAZ GSM 200) measured the air temperature and relative humidity once per hour. From these double measurements an hourly average per location for both temperature and humidity was calculated. Wind speed and direction at 2 m height was recorded every 10 min by an anemometer (Davis Vantage Pro II) and stored as hourly mean and maximum. Additionally, hourly sums of precipitation were collected by a rain gauge (accessory of CAPAZ GSM 200). At each mason bee study location, the air temperature and relative humidity were collected by a validated data logger (Gemini TGP-45000) at 30 cm height which was protected against rain and direct sunlight. Similar to the honey bee study locations, an anemometer was set up at each mason bee study location to measure wind speed and direction. Daily sums of rainfall for all mason bee locations were obtained from the German Weather Service (DWD) of a local weather station at Goldberg, approximately 10 km east of the test site.

The measured weather conditions at the study locations coincided with official measurements (Statistisches Amt Mecklenburg-Vorpommern 2015) indicating a warm and relatively dry period during the third pentad of April, followed by lower temperatures at the beginning of May which increased again towards the end of May. Rainy periods were concentrated during the second and third pentads in May (Statistisches Amt Mecklenburg-Vorpommern 2015). In general, no weather extremes occurred during the exposure phase. Although they are not representative for the whole study area, weather data collected at the honey bee study locations were analysed for differences between the study sites. There were no significant differences between the study sites in the daily mean temperature, the daily mean of relative humidity, the daily sum of precipitation, and the daily mean wind speed (Table 2, Fig. S1).

#### Agronomic practice

Information about agricultural practices at the study fields, such as treatment with other PPPs and their application rates was gathered from the farmers for the period of the monitoring study as well as additional details of the variety, drilling rate, and origin of OSR seeds. Apart from the seed dressing and the request not to apply any further neonicotinoids between drilling in August 2013 and harvest in July 2014, the farmers were allowed to decide for themselves about all agricultural practices including the application of other PPPs.

The study was conducted with the cooperation of independent farmers who made the decisions about all agricultural practices, the seed types and the PPP applications and so there was some variation between study fields. In order to comply with local conditions and to optimally schedule agricultural activities, several OSR varieties were used. In total, 33 different OSR varieties were drilled at the study fields of which the most common were *Genie* (R: 40.6 % of crop area, Rapool-Ring GmbH), *Sherpa* (R: 25.3 %, T: 22.2 %, Rapool-Ring GmbH), and *Xenon* (T: 21.4 %, Rapool-Ring GmbH). The diversity of OSR varieties was larger at the test site mainly because the study field T13 was used for a variety demonstration and, thus, contained 22 different varieties sown in stripes each less than 1 ha in size. Two of these demonstration varieties were dressed with thiamethoxam (3 g/kg seeds) instead of clothianidin. The test fields T1, T9, T10, T14 and T15 also contained more than one OSR variety (Table S1). Grouped by their anticipated time of flowering, early varieties dominated at the test site while intermediate flowering varieties dominated at the reference site (Table S1). However, the difference in the period of full flowering between early and intermediate varieties constitutes 3–4 days only and nectar and pollen are available beyond that period. Furthermore, small scale microclimatic conditions may cause a higher variability in the flowering time of OSR. The seeds had an average thousand seed weight (TSW) of 6.2 ± 1.3 g. The General Linear Model revealed that the TSW differed due to the OSR variety (F_31, 32_ = 2.42, *p* = 0.007) and the amount of seed dressing (F_1, 32_ = 5.39, *p* = 0.027), but not between the reference and test sites (Table 2). The drilling rate of OSR seeds averaged 3.4 ± 1.1 kg/ha and was significantly higher in study fields at the test site compared to the reference site (Table 2, Table S1). However, compared at the landscape level and weighted by the field size of study fields, the drilling rate did no longer differ between the treatments (Table 2). Based on differences in the TSW and the drilling rate, the average number of seeds per square meter was significantly higher at test fields compared to the reference fields (Table 2).

During the development of the OSR plants, they received on average 4.8 ± 0.4 and 4.1 ± 0.9 insecticide spray applications at the reference and test site, respectively. This difference was statistically significant (Table 2) and was due to a significantly higher number of applications in autumn at the reference site (Table 2). This was because the OSR plants lacked the insecticidal seed treatment and most of the study fields at the reference site received an additional pyrethroid spray treatment in autumn 2013 to control cabbage stem flea beetles (*Psylliodes chrysocephalus*) and cabbage root fly (*Delia radicum*). The number of additional insecticide applications in spring 2014 did not differ statistically significant between the sites. The most frequently applied compounds were the pyrethroids etofenprox (Trebon 20 EC®) and beta-cyfluthrin (Bulldock®). The oxadiazine indoxacarb (Avaunt®) which is classified as harmful to honey bees and bumble bees when exposed to direct treatment (DuPont 2004; van der Steen and Dinter 2008), was applied at seven reference fields in March and the beginning of April 2014. This was well in advance of the establishing of the bumble bee hives at the study locations (by at least 2.5 weeks). Pymetrozine (Plenum®), the only triazine used on the study fields, was applied at T7–T10 and T13–T15 at least 3 weeks prior to the start of the exposure phase of the bees.

The agronomic practice of non-OSR fields at the study sites are also not expected to have any confounding effect on this study. Although sowing of maize fields overlapped with the flowering of OSR and the dust from sowing operations of neonicotinoid dressed maize was shown to adversely affect honeybees under specific exposure conditions (Pistorius et al. 2009), neonicotinoid dressings of maize are not authorized in Germany since 2008 (Federal Office of Consumer Protection and Food Safety 2009) and the sown maize only contained a fungicide or no dressing at all (personal communication with farmers). Furthermore, due to the restrictions on neonicotinoid use since December 2013 (European Commission 2013), confounding effects of neonicotinoid applications at adjacent fields can also be excluded.

#### OSR development

At the study fields in the core area, the development of the OSR crops was surveyed seven times between November 2013 and the end of the exposure phase in May 2014. Corresponding BBCH-stages were determined based on the adjusted code for OSR development (Federal Biological Research Centre for Agriculture and Forestry 2001). The rate of emerged OSR plants and the respective plant density was assessed prior to the stem elongation in March 2014. For methodological details of the density estimation see Rolke et al. (2016b).

Drilling of the OSR seeds took place between 13 and 29 August 2013 with a peak on 18 and 19 August 2013 which was similar for study fields at the reference and test sites (Table 2). The rate of plants surviving the winter averaged 68 ± 25 % and was equal at study fields of the reference and test sites (Table 2). However, due to the differences in the drilling rate, the OSR plant density was higher at test fields compared to reference fields (Table 2). The OSR crops at the study sites developed homogenously across all seven assessments based on the BBCH stages (Table 2). By the first assessment on 21 November 2013, almost all OSR plants had reached BBCH stage 19 (“9 or more leaves unfolded”). More importantly, a few days before the start of the exposure phase of the bees (21 April 2014) at least 30 % of flowers were open on all study fields (BBCH 63), ensuring sufficient food was available for the bees. Full flowering of OSR (BBCH 65) lasts for three to five weeks. Accordingly, by 22 May 2014 flowers at the majority of study fields had withered and only 5 % of the plants were still with flowers. The exposure phase was terminated at this stage because the OSR plants did not provide sufficient amounts of nectar and pollen for foraging bees any more.

The OSR yield standardised to the field size was significantly higher at the test site than at the reference site (Table 2). This difference was in line with yield differences in previous years probably due to a slightly more productive soil in the south of the study area. This difference could also have been due to early losses of OSR plants at the reference site which lacked the neonicotinoid seed treatment although it was compensated for by the additional pyrethroid spray application. The lower plant density at the reference fields may also have contributed to the difference in yields. However, the different plant densities did not affect the availability of OSR nectar and pollen for the investigated bees because the coverage of OSR at the study sites provided food in excess and all bee hives developed very well during the exposure phase (Rolke et al. 2016a; Sterk et al. 2016). Furthermore, the average yields at both study sites were close to the average yield of winter OSR of 37.5 dt/ha in the district of Ludwigslust-Parchim in 2014 (Statistisches Amt Mecklenburg-Vorpommern 2015).

In summary, the environmental and agronomic conditions at the reference and test site were largely similar with the exception of the insecticide treatment. Thus, local environmental conditions were considered not to have any significant confounding effect on the results of the monitoring study.

### Crop fidelity of bees

Three measures were applied to ensure that the investigated bees foraged at the OSR that was grown from seeds either treated with or without a clothianidin dressing. Firstly, the study sites were selected to provide a high density of OSR crops but did not include any other mass flowering crop which was suitable as bee forage during OSR flowering. Furthermore, as described above, the size of the study sites was intended to cover the foraging flights of the investigated bees.

**Fig. 3:**
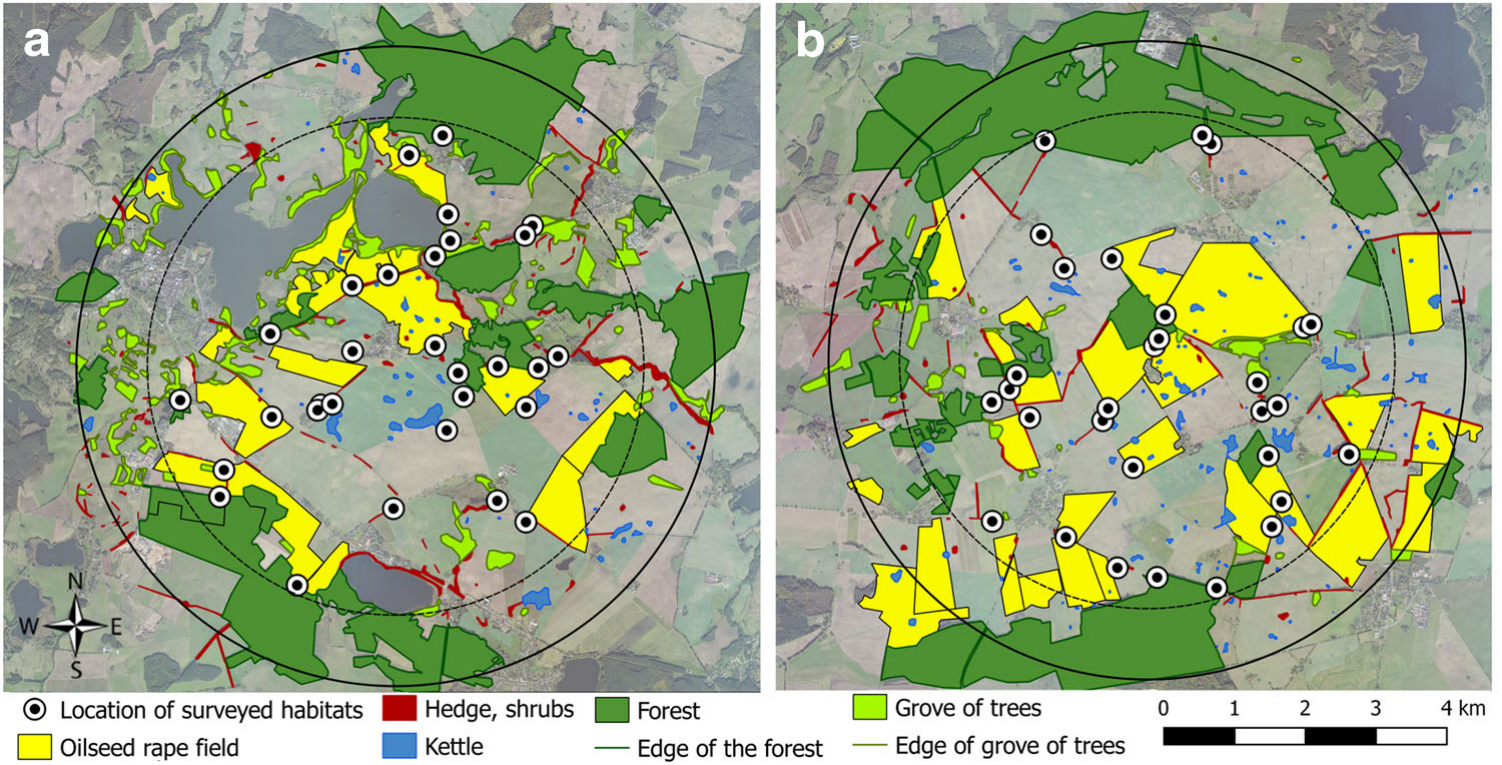
Sampling sites (black points) and occurrence of habitats with alternative forage for bees at the reference (**a)** and test site (**b)**

Secondly, bees may also use weeds and flowering plants at field margins, forest edges, settlements and grasslands as pollen and nectar resource apart from cultivated crops (Stanley et al. 2013a; Stanley and Stout 2014). Accordingly, a detailed assessment of the abundance of alternative bee forage (pollen and nectar provided by other than OSR plants) during OSR flowering was obtained by a semi-quantitative survey of non-crop habitats at both study sites. During OSR flowering, 10 representative hedges, kettles, and forest edge habitats were visited once and the abundance of flowering plants assessed along a transect of at least 150 m in length (Fig. 3). Taking the variations in importance for the different bee species into account, each flowering plant species was rated on an ordinal scale as following: 0—no occurrence of plant species; 1—very few flowers present, to be neglected as food source for bees; 2—few flowers present, sufficient as food source for individual bees; 3—numerous flowers present, sufficient as food source for bees; 4—abundant flowers present, sufficient as food source for bees, very attractive. A similar classification was carried out for grasslands and field margins from photographs taken during OSR flowering. Ratings of all plant species present at a sampling site were averaged and used to calculate a mean for each habitat type per study site. The resulting value per habitat was weighted by the area of the habitat according to Eq 1.

1 $${\mathrm{Availability}}\,{\mathrm{per}}\,{\mathrm{habitat}} = \frac{{{\sum} {{\mathrm{mean}}\,{\mathrm{rating}}\,{\mathrm{per}}\,{\mathrm{sampling}}\,{\mathrm{site}}} }}{{{\mathrm{number}}\,{\mathrm{of}}\,{\mathrm{sampling}}\,{\mathrm{sites}}}} \\ \times {\mathrm{area}}\,{\mathrm{of}}\,{\mathrm{habitat}}$$

**Fig. 4:**
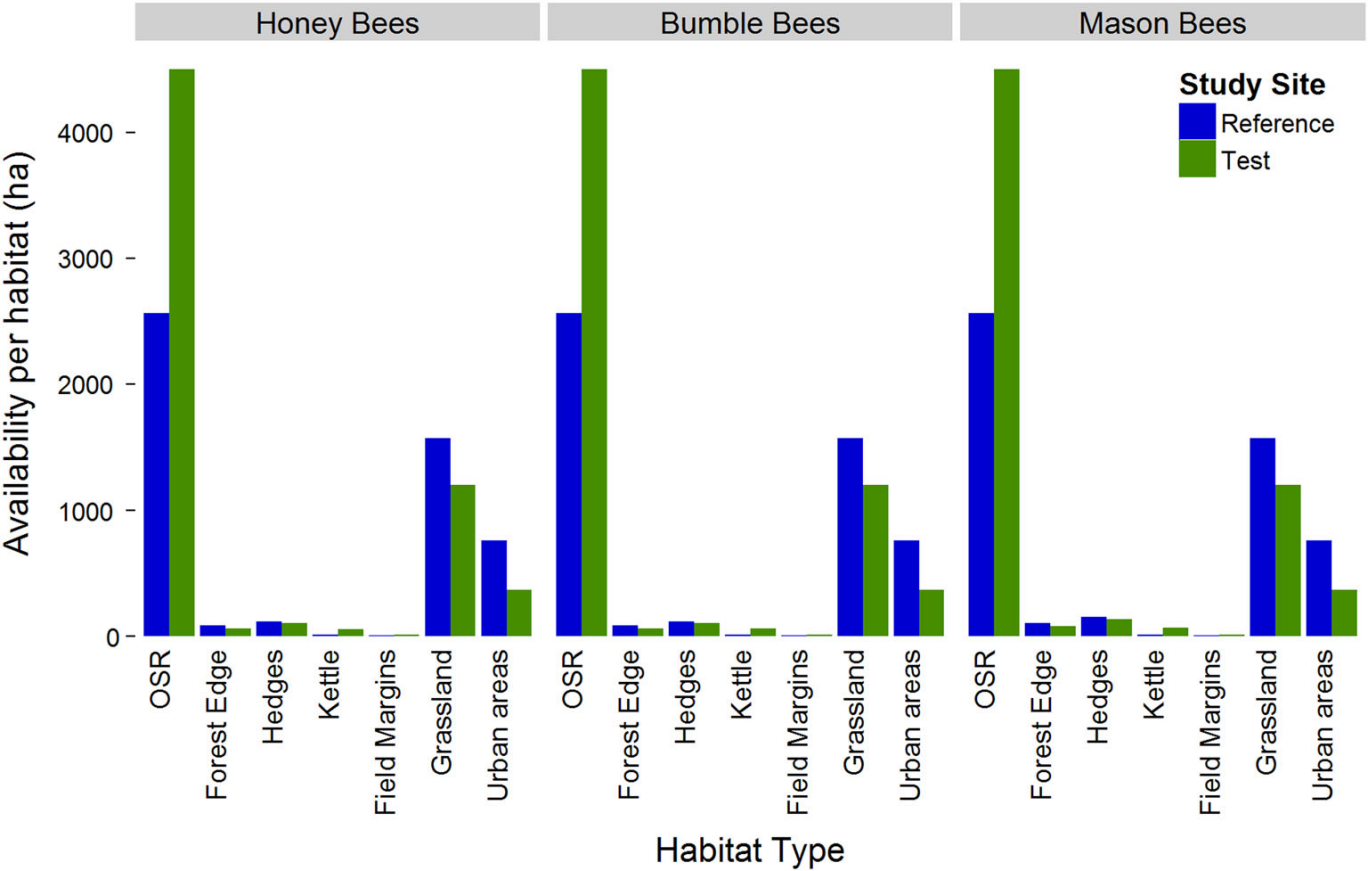
Availability of alternative forage plants at different non-crop habitats of the reference and test site for the three bee species in focus (*Apis mellifera*, *Bombus terrestris*, and *Osmia bicornis*). The availability is calculated from the rating of attractiveness and availability of several plant species and the size of the area covered with the respective habitat

In total, 38 plant species were found in hedges, kettles and forest edges which are attractive to bees during OSR flowering and may have represented a forage source for at least one of the bee species studied. Of these plants, 7.2 species occurred on average per surveyed site. The Wild Chervil (*Anthriscus sylvestris*), Common Oak (*Quercus robur*), Archangel Fair (*Lamium album*), and Dandelion (*Taraxacum spec*.) were relatively common and occurred at more than half of the surveyed sites. Only trees and shrubs were highly available as food resource for foraging bees. Hedges were on average more diverse than kettles and forest edges. For grassland, field margins and urban areas the coverage with flowers suitable as bee forage were estimated to constitute 10 % which is transformed to a rating of 1.5. Compared to OSR, which is highly attractive at least to honey bees and available at 13.9 % of the area of the study sites, the alternative foraging resources play only a minor role (Fig. 4).

However, this assessment of alternative forage for bees at non-crop habitats can only give an approximate estimate of the availability of alternative food resources. Therefore, a third approach to ensure that the investigated bees fed on the OSR of the study sites, was to analyse the composition of the pollen collected by the honey bees, earth bumble bees and red mason bees, as well as the nectar and honey collected by honey bees. For methodological details see the respective papers in this issue (Peters et al. 2016; Rolke et al. 2016a; 2016b; Sterk et al. 2016). This investigation confirmed that all three bee species foraged on OSR although to different degrees. In particular red mason bees collected pollen from a diversity of plants available in the close vicinity of the study locations. Nevertheless, the exposure of the bees to OSR at the study site was proven and the difference in the amount of OSR among the utilized food resources reflect the typical exposure of the different bee species to OSR under natural field conditions.

### History of study fields

#### Crop and PPP history of study fields

The study area has a long agricultural history which might contribute confounding effects on the monitoring study because PPPs from agricultural applications seem to occur ubiquitously in the environment (Stewart et al. 2014, Cutler et al. 2014). Hence, the difficulty arises to find control sites which coincide with the test sites in their background levels off PPPs without confounding the study results (e. g., Cutler et al. 2014; Rundlöf et al. 2015). In order to reduce the possibility that PPPs applied to the study fields in recent years might have affected the outcome of the monitoring study, detailed information on the agricultural practice at the study fields within the five years previous to the monitoring, including cultivated crops, treatment with PPPs and their application rate were obtained from the farmers.

**Fig. 5:**
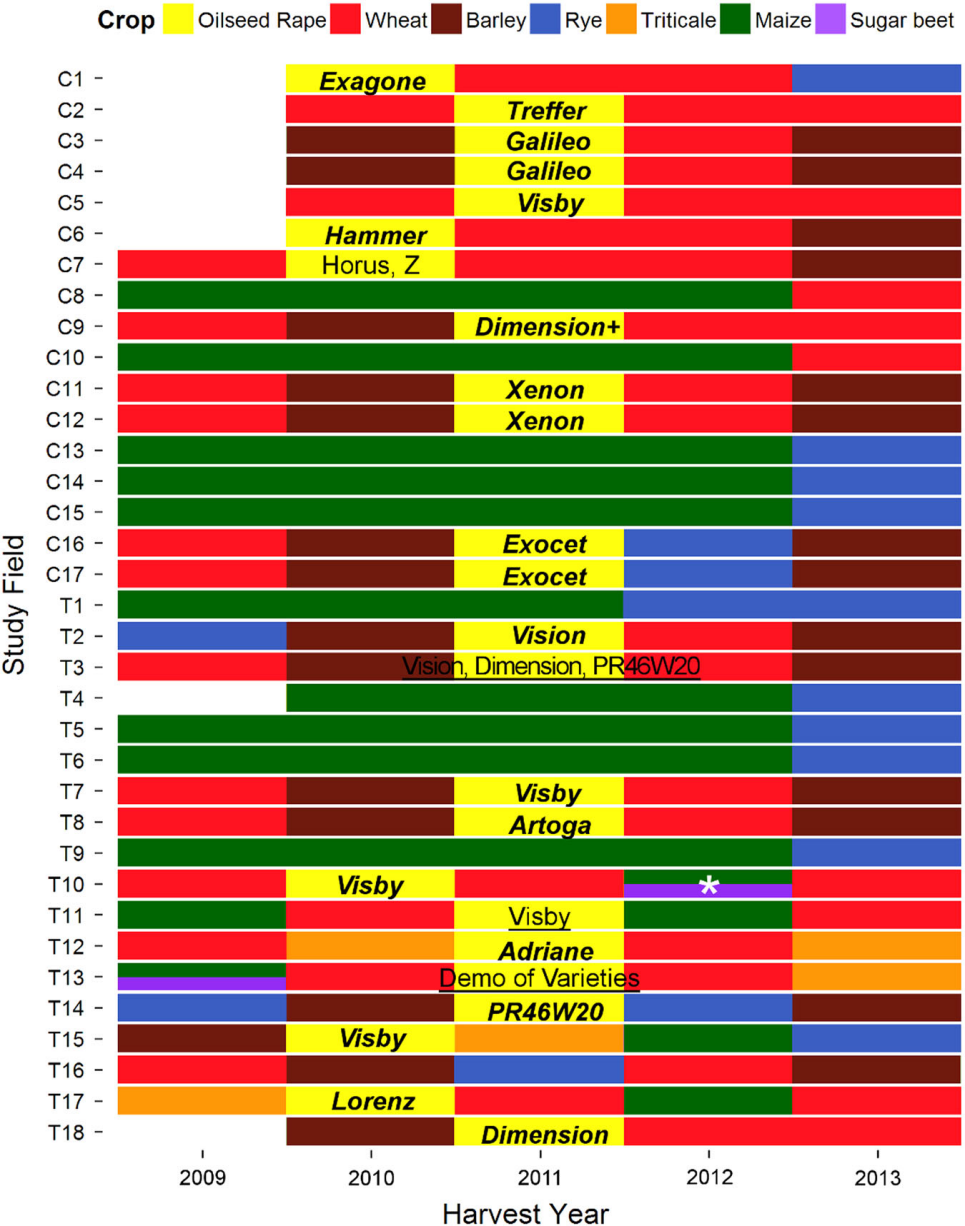
Crops of the study fields in the five years previous to the study. Two colors at one field indicate a field which was partitioned to cultivate two different crops. For some study fields no data were available for 2009, these plots were left blank. OSR varieties given in ***bold italic*** indicate a dressing with Clothianidin. For underlined OSR varieties no information on seed dressing was available. Further neonicotinoid seed dressings were used at the sugar beet (*)

The crop history of the study fields between the harvest years 2009 and 2013 indicated common crop rotations following good agricultural practices at both reference and test site (Fig. 5). The most common crops cultivated at the study fields during the previous five years were wheat and maize. The previous OSR cultivation at the study fields dated back between three and more than five years. At least 83.3 % of previously cultivated OSR contained a clothianidin dressing, and for 12.5 % of the former OSR cultivated there was no information of seed dressing available. Seed dressings of crops other than OSR were primarily fungicides and did not contain clothianidin or other neonicotinoids as an active ingredient except for sugar beet cultivated at the test field T10 in 2012 which was dressed half with clothianidin and half with thiamethoxam, a neonicotinoid of which the primary metabolite is clothianidin (Nauen et al. 2003). Insecticides applied to the study fields during the five years previous to the study were mainly pyrethroids (70 % of insecticide applications) and neonicotinoids (21 %). Oxadiazines (5 %), carbamates (2 %), and organophosphates (2 %) played only a minor role. Neonicotinoids were primarily applied as seed dressings of which 96 % contained clothianidin and 4 % thiamethoxam. Thiacloprid accounted for 96 % of all neonicotinoid spray applications whereas acetamiprid was applied once. Imidacloprid had not been applied during the last five years. At all study fields, the last applications of neonicotinoids were in 2011 or earlier, hence, at least 3 years prior to the monitoring study.

#### Residues in soil before drilling

The collected soil samples were analysed for clothianidin residues because of the predominant use of this active ingredient and its relatively long half-life in soil (Krupke et al. 2012). Based on the information gained by the agricultural practices of the farmers, the analysis of the soil was restricted to clothianidin because other neonicotinoid insecticides were not applied or the application dated back a multiple of the respective half-life. Samples of the test field T18 were only taken after drilling of OSR seeds and therefore were not suitable for the analysis of background clothianidin residue levels. Preparation of soil samples was based on QuEChERS methods (“Quick Easy Cheap Effective Rugged Safe”, DIN EN 1552 (2008); Lehotay 2006). To determine clothianidin concentrations, liquid chromatography coupled with tandem mass spectrometry (LC-MS/MS) was applied. The chromatographic system used was a high performance liquid chromatograph with a reversed phase chromatography (Zorbax Eclipse C18, 50 × 2.1 mm, 1.8 μm column) coupled with tandem mass spectrometry and electrospray ionisation (AB Sciex API 6500 Triple Quadruple Mass Spectrometer, Analyst version 1.6.2). The coefficient of determination for calibration curves was above 0.996. In soil, the limit of quantification (LOQ_soil_) was 5 μg/kg dry weight soil and the limit of detection (LOD_soil_) was 1.5 μg/kg.

In 82 % of the 134 samples no clothianidin was detected (<LOD_soil_), whereas residues below the LOQ_soil_ were found in 6 and 18 plots of study fields at the reference (R6, R7, R9, R12) and test sites (T3, T4, T7, T8, T13, T14), respectively. Calculations based on the upper limits of LOD_soil_ and LOQ_soil_ revealed a very conservative estimate for average soil residues of 2.1 ± 1.3 μg/kg which did not differ significantly between the study sites (Table 2). This residue concentration is in the range of clothianidin residues reported for agricultural soils with repeated drilling of clothianidin dressed corn (2–11.2 μg/kg (de Perre et al. 2015), 2013: 4.0 ± 1.1 μg/kg, 2014: 5.6 ± 0.9 μg/kg (Schaafsma et al. 2015), 7.0 ± 4.2 μg/kg (Xu et al. 2016)) and OSR seeds (5.7 ± 4.0 μg/kg (Xu et al. 2016)). The distribution of plots with clothianidin residues among the study fields did not reveal any correlation with the crop or PPP history during the previous five years. Since clothianidin is known for its ageing behaviour in soil and has also a low bioavailability of 6–10 % (Xu et al. 2016), the non-quantifiable soil residues on 18 % of all sampled plots were considered not to contribute to translocation into bee-relevant matrices (nectar and pollen). This assumption has been confirmed by the later residue findings in nectar and pollen samples on the reference sites under confined conditions (>90 % of all residue samples not detectable at LOD_nectar/pollen/honey_ = 0.3μg/kg).

Because crop history and PPP applications resembled each other at the two study sites and based on the applied PPPs no adverse long-term effects are to be expected.

### Ensuring exposure to focal PPP

#### Clothianidin loading of OSR seeds

Another uncertainty in field studies is whether the investigated free flying bees are exposed to the PPP under consideration. To be able to quantify the potential exposure, OSR seed samples taken before drilling were analysed for their clothianidin loading. Before sowing, samples of approximately 500 g were taken from all OSR seeds for the analysis of clothianidin loadings. The seed samples were pre-processed by mixing with an acetonitrile/water solvent mix (4/1, v/v) to extract the clothianidin and analysed by liquid chromatography coupled with tandem mass spectrometry similar to the analysis of residues in soil samples described above. The coefficient of determination for calibration curves was again above 0.996. The LOQ_seeds_ of clothianidin residues in OSR seeds was 1.0 mg/kg.

The amount of clothianidin in the seed coating of treated OSR seeds averaged 7.8 ± 1.5 g/kg and ranged between 43.9 % and 108.2 % of the nominal concentration of 10 g/kg (Table S1). Traces of clothianidin were also found in the OSR seeds of the reference site with a median loading of 0.02 g/kg (range 0.001–0.226 g/kg). These low amounts of clothianidin arose from residues in commercial facilities for seed treatment. Though OSR seeds used on the reference site were not treated with clothianidin, the seeds were processed in common seed treatment facilities for dressing with the fungicides thiram and dimethomorph. The study fields R17 and R18 at the edge of the reference site (Fig. 1) also contained a clothianidin seed dressing. However, this did not diminish the value of the monitoring study because the nearest study locations of bees were 2.9 km apart (compare Rolke et al. 2016b) and the Great Sternberg Lake in between most likely formed a natural barrier for the bees. Accordingly, the lack of clothianidin residues in pollen and nectar from the closest study locations verified that the bees at the reference site did not forage on these two study fields where the seeds had been treated with clothianidin (Rolke et al. 2016b) probably due to the ample food available in the vicinity of the hives.

Based on the clothianidin-loading of seeds and drilling rates, on average 28.8 ± 10.0 g/ha of clothianidin were applied to the study fields of the test site and 0.19 ± 0.25 g/ha at the reference site during drilling. If we assume an equal distribution of OSR seeds and clothianidin at the field and an average soil density of 1.5 kg/L, the clothianidin concentration at the test and reference site amounted to 19.2 ± 6.7 μg/kg and 0.13 ± 0.17 μg/kg, respectively, in the uppermost 10 cm of the soil after drilling. The contamination at the reference site is below the average residue concentration in the soils before drilling and, thus, considered very unlikely to have a confounding effect on the study results.

#### Clothianidin residues in bee forage

To further verify the exposure to clothianidin of the bees at the study locations, pollen collected by honey bees, earth bumble bees and red mason bees as well as nectar and honey collected by honey bees were analysed for clothianidin residues and its metabolites thiazolylmethylurea and thiazolylnitroguanidine. For methodological details see Rolke et al. (2016b). In an additional semi-field tunnel-tent study (bees confined to the test crop in insect-proof cages at all study fields), nectar and pollen samples were collected from honey bees foraging exclusively on OSR. The residues in pollen and nectar (N_pollen_ = N_nectar_ = 39) from the tunnel-tent study indicated a clear exposure to the neonicotinoid at the test site, whereas at the reference site no residues were detectable in the majority of samples (96 % for pollen, 100 % for nectar, N_pollen_ = N_nectar_ = 34).

Similar results were obtained for the investigated bee species at the study locations which could freely forage. Neither clothianidin nor its metabolites were detected (LOD_nectar/pollen/honey_ = 0.3 μg/kg) in any pollen sample collected by honey bees (*N* = 96), bumble bees (*N* = 6) and mason bees (*N* = 6) at the reference site, whereas a few nectar samples (5.6 %, *N* = 96) and 62.5 % of honey samples (*N* = 48) contained non-quantifiable amounts (LOQ_nectar/pollen/honey_ = 1.0 μg/kg) of clothianidin. In contrast at the test site, clothianidin residues were detected in the majority of pollen (N_honey bee_ = 96, N_bumble bee_ = 6, N_mason bee_ = 6), nectar (*N* = 96) and honey samples (*N* = 48), mainly at concentrations below the LOQ_nectar/pollen/nectar_ but also at clearly quantifiable concentrations with a maximum of 2.7 μg/kg in pollen, 1.6 μg/kg in nectar, and 2.1 μg/kg in honey (Rolke et al. 2016b). These results clearly demonstrate that the investigated bees were exposed to clothianidin while foraging on OSR grown from clothianidin dressed seeds at the test site.

#### Residues in soil after harvest

The half-life of clothianidin in soil was reported to range between 13.3 and 305.4 days under field conditions (mean: 120.1 days, European Commission 2005). In order to assess the persistence of clothianidin in the soil of the study area after applying a known amount of the neonicotinoid as seed dressing, the central study fields T7, T8, and T10 of the treatment site were sampled again after the harvest of OSR plants in August 2014. This time, one soil sample was taken per subplot (*N* = 15). The analysis of clothianidin residues was conducted as described above for the previous analysis of clothianidin residues in soil. The residues of clothianidin were below the LOQ_soil_ (5 μg/kg) in 11 of the 15 samples and the maximum concentration found was 5.9 μg/kg at study field T10. These concentrations are considerably lower than the amount of clothianidin in the soil after drilling which was calculated as above to constitute 15, 9.4 and 16.9 μg/kg on the study fields T7, T8 and T10, respectively. The analysed residue concentrations indicate clothianidin concentrations to dissipate by 50 % in 0.5 years or less which corresponds well with the reported dissipation of clothianidin in agricultural soil (Schaafsma et al. 2015).

## Representativeness of the study area

The validity of results of the large monitoring study needs to be considered for regions other than where the study was conducted. Therefore, an assessment of whether the study area is representative in terms of the eco-physiological climate was included, the OSR phenology, and the land use on a regional scale, that is the district of Ludwigslust-Parchim (Fig. 1a), as well as for the major OSR cultivation areas in Germany. For this analysis, the inner core of both study sites, encompassing the inner 7 km in diameter each (Fig. 1b), were investigated in depth because the majority of studied bees was considered to forage in this area of 2 km around the bee hives and nesting shelters (Gathmann and Tscharntke 2002; Steffan-Dewenter and Kuhn 2003; Walther-Hellwig and Frankl 2000). Furthermore, a comparison was conducted between the study area and other regions with OSR cultivation at the European level based on the density of OSR cultivation and the availability of alternative bee forage, to facilitate the transferability of the results.

### Climate and OSR phenology

The eco-physiological climate classification as developed by Lauer et al. (2002) was applied. This classification is based on empirical data of the heat and water budget of a region and also integrates interactions of the “climate-vegetation-soil” system. The climate of the study area was classified as Cmhα, which describes a warm temperate climate (C) with a mesotherm (m) 5–6 months lasting thermal growing season and a humid (h) 7–9 months lasting hygric growing season, as well as a high maritime degree of continentality (*α*). The Cmhα climate predominates in the district of Ludwigslust-Parchim as well as in Mecklenburg-West Pomerania. Only in the very south of the federal state outside the study area does the climate change to sub-humid (sh), a 5–6 months lasting hygric season and a submaritime degree of continentality (*β*).

Considering the weather parameters individually for the period of winter oilseed rape flowering, data obtained from the European Centre for Medium-Range Weather Forecasts were analysed (Table 1). In the study area, this period is characterized by mean temperatures of 11.8 °C (minimum 6.6 °C, maximum 17.3 °C), a precipitation sum of 54.2 mm, and a solar radiation sum of 566 MJ/m^2^ (Fig. S3). This is consistent with the long-term average weather conditions of the entire Ludwigslust-Parchim district with a mean temperature of 12.1 °C (minimum 6.7 °C, maximum 17.7 °C), 53.5 mm precipitation and 561 MJ/m^2^ solar radiation during OSR flowering. Similarly, the weather conditions in Mecklenburg-West Pomerania during OSR flowering are characterised by a mean temperature of 11.8 °C (minimum 7.3 °C, maximum 16.5 °C), 47.9 mm precipitation and 580 MJ/m^2^ solar radiation. There is only little deviation towards the Baltic Sea Coast (Figs. S2, S3). Thus, with deviations in all average temperatures of less than 1 K, a difference in the 30-day precipitation sum of only 6.3 mm and 14 MJ/m^2^ in the solar radiation, the study area can be seen as representative also for the federal state of Mecklenburg-West Pomerania in terms of the weather conditions during OSR flowering.

The average air temperature during OSR flowering for the years 2000–2013 was further used to calculate temperature sums and growing degree days for the analysis of OSR phenology. The flowering period of OSR is defined as starting when the first flowers open (BBCH 60, Federal Biological Research Centre for Agriculture and Forestry 2001) and ends when all petals have fallen (BBCH 69). Typically, in Central Europe the flowering of winter OSR lasts 3–5 weeks, depending on the weather conditions (German Weather Service 1995–2014). Therefore, the average duration of flowering was set to 30 days for further calculations. The analysis revealed that in the study area flowering of winter oilseed rape starts on average in the third decade of April (between 21st and 30th April) and ends on average between the 11th and 20th May, hence, in the second decade of May. According to the similarity in climate, these dates also apply to the flowering of winter OSR in general in the district of Ludwigslust-Parchim (average OSR flowering third decade of April until second decade of May) and the majority of OSR cultivation areas in Mecklenburg-West Pomerania (average OSR flowering third decade of April/first decade of May until second/third decade of May).

### Land cover and land use

In order to estimate the availability of OSR and other mass flowering crops as bee food, the land cover of different habitats and crops was assessed as described above. Comparable land use data at the level of the district and the federal state were taken from agrostatistical figures for the harvest year 2014 (Statistisches Amt Mecklenburg-Vorpommern 2015) and regional statistical databases of the surface area according to actual use for 2012 (Statistisches Amt Mecklenburg-Vorpommern 2012). Europe-wide data on the amount of OSR cultivation per administrative unit (Nomenclature des unites territoriales statistiques—NUTS, level 2 and 3) were obtained from official national statistical services for the years 2010–2013, and those for other land use types were derived from CORINE 2006/2010 land use data provided by the European Environment Agency, Copenhagen.

For the study area, the analysis of LULC revealed that 60.9 % of the 77.0 km^2^ comprising the inner cores of the study sites were covered with arable land. Winter OSR was cultivated on 1406.2 ha (30.0 %) of the agriculturally used area in the harvest year 2014, which constitutes 18.3 % of the core area of the study sites (compare Fig. 2). Other crops cultivated in the study area during the monitoring study were different grains (32.9 % of core area), maize (8.6 %) and sugar beet (1.1 %), which are all not suitable as forage sources for bees during OSR flowering. At the local (district) and regional (federal state) scale, arable land covered 43.5 and 46.4 % of the landscape, respectively, and winter OSR is cultivated at 7.0% and 11.5 % of the area, respectively. Grains, maize, and sugar beet were cultivated at 19.4%, 10.0% and 0.3 % and 23.3 %, 6.1%, and 10.7% of the area of the district and federal state, respectively. Grassland covered equal amounts of 11.2%, 12.4%, and 11.1 % of the core area of the study sites, the district Ludwigslust-Parchim, and Mecklenburg-West Pomerania state, respectively. Urban areas covered 5.6 % of the core area of the study sites which is in the same range as for the district (7.3 %) and federal state (8.0 %). Forests constituted 28.1% and 21.8% of the district and federal state, respectively, but considerably less in the study area (10 %) which is due to the selection criteria for the study area of high coverage with arable land and OSR crop in particular.

Exclusively winter OSR was cultivated in the study area, but no summer OSR. This is consistent with the higher economical importance of winter OSR and the negligible percentages of summer OSR in Germany (2014: 0.3 % of OSR cropping area, Federal Statistical Office) and the European Union (2014: 6.5 %, Eurostat).

**Fig. 6:**
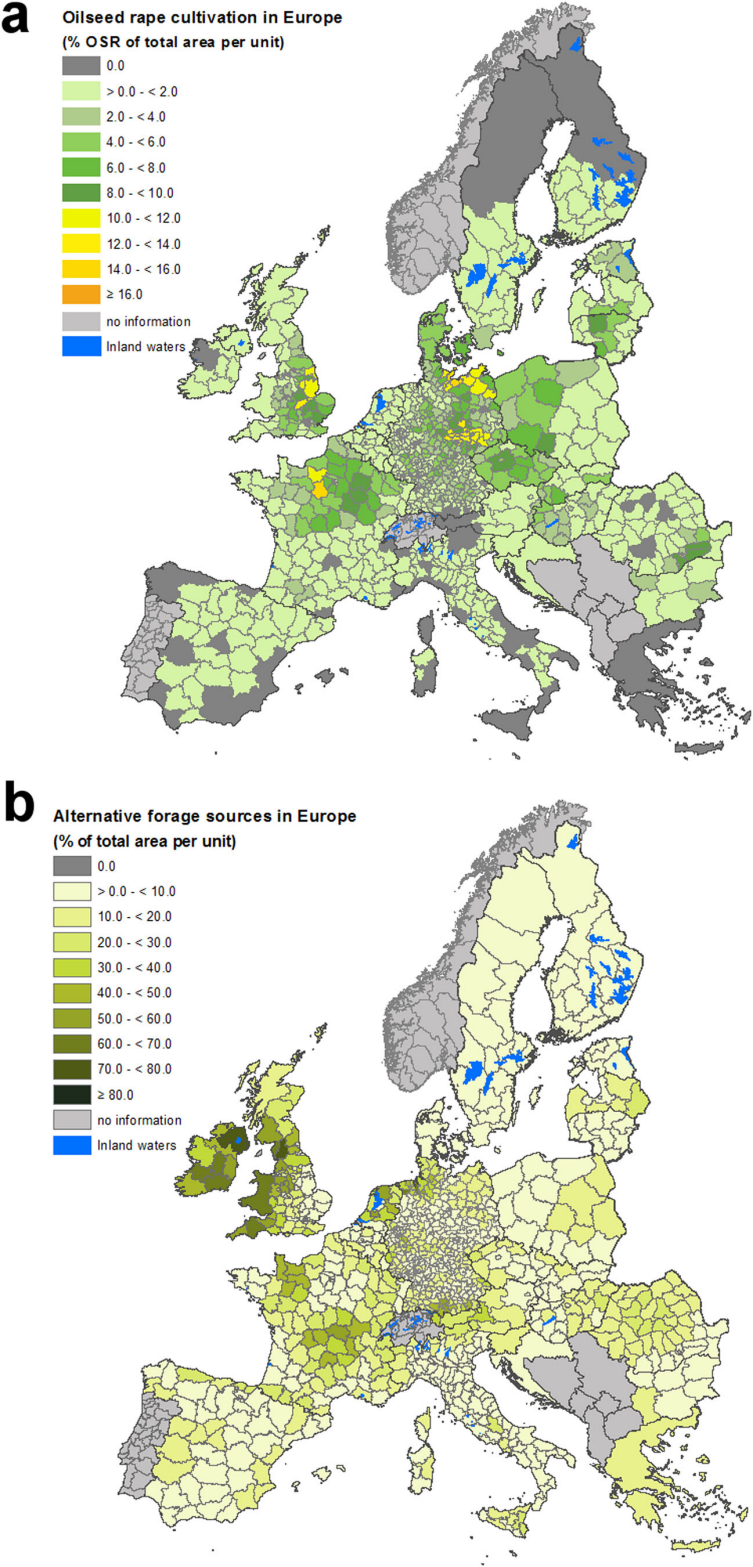
Spatial distribution of regional OSR cropping density (**a)** and coverage with land use types providing alternative forage plants for bees (**b)** across Europe

Compared to other OSR cultivation areas in Europe, equally high OSR densities as in the study area of above 16 % of the area are hardly found at the larger scale of the administrative units (Fig. 6a). Nevertheless, considering the average OSR density of Mecklenburg-West Pomerania of 11.5 %, it is still at the upper range of OSR densities at administrative levels in Europe, with only few areas having similar or higher densities (Fig. 6a).

### Alternative bee forage

During flowering, OSR is a highly attractive food resource for bees (Abrol 2007, Holzschuh et al. 2013, Stanley et al. 2013b). Nevertheless, the polylectic bees may also forage from other crops and flowering plants. LULC categories providing high amounts of alternative bee forage during OSR flowering were fruit trees and berry plantations, forest margins, natural grassland, and pastures. For these LULC categories, the relative coverage per administrative unit were calculated for the OSR cultivation areas in Europe (Fig. 6b). In the district Ludwigslust-Parchim, alternative bee forage habitats as defined above cover 15.2 % of the area. This is consistent with almost all regions of high OSR density having a relatively low amount of habitats with alternative bee forage. The highest percentages of alternative forage habitats exist in regions with large areas of grassland, as found in Ireland, Wales, the northwest and very south of Germany, The Netherlands, as well as the Normandy and the Central Massif in France (Fig. 6b). However, these regions in turn contribute little to the overall OSR cultivation (Fig. 6a).

Therefore, at the smaller scale of the study area, the land cover categories of arable land and OSR crop occur at a high density due to the selection criteria applied to identify a suitable study area. Accordingly, in terms of OSR density and lack of other mass flowering crops during OSR flowering, the study area constitutes a worst case in terms of high exposure to OSR for foraging honey bees considering actual field conditions. Furthermore, there are no major differences in climate, OSR phenology and land use between the study area, the district Ludwigslust-Parchim and Mecklenburg-West Pomerania state. Therefore, the study area can be seen as representative for the major winter OSR cultivation region in Germany whereas in the European context, at a broader scale, OSR is usually cultivated at lower densities and the availability of alternative bee forage in arable fields and other habitats is higher. Hence, the study area constitutes a worst case of high OSR exposure with few alternative food sources also at the European level for honey bees and to a lower extend also for earth bumble bees because they can profit from more limited food resources. Generalizing the study results from the investigated red mason bees to the European level and other solitary bees is, however, limited due to the diversity of species and their different life styles.

## Conclusion

Monitoring studies at the landscape level are difficult to conduct, complex, expensive and many aspects have to be considered to avoid confounding effects. This is probably the reason why they are not often performed in the context of regulatory risk assessments of PPPs to pollinating insects. In general, few of these studies have been conducted so far to investigate the side-effects of crops treated with neonicotinoids on pollinating insects under realistic field conditions (Godfray et al. 2014). Those that have been conducted (e.g. Cutler et al. 2014; Pilling et al. 2013; Rundlöf et al. 2015; Thompson et al. 2013) were criticized amongst other things for their low statistical power due to limited replication, contaminated control sites and further differences between environmental conditions at the control and treatments sites. This study aimed to avoid all these confounding effects in this large-scale monitoring study of side-effects of clothianidin seed-dressed OSR on three different pollinator species. This paper presents the implementations of the identified requirements for the study.

The study sites were shown to be sufficiently large to ensure the exclusive exposure of the investigated bees to the conditions controlled for at the respective study site. An alternative approach would have been to compare several paired sites distributed over a larger spatial scale as for example done by Rundlöf et al. (2014, 2015), and Cutler and Scott-Dupree (2014). However, this approach would have added considerably to the amount of natural variability and, hence, limited the statistical conclusions possible.

In addition, the environmental conditions of the reference and test sites were shown to be as similar as possible under natural conditions and possible confounding effects will have been reduced to an absolute minimum. Therefore, we conclude that the results of the different bee monitoring studies conducted in this project are valid and not significantly influenced by environmental conditions.

The investigations reported here verify the suitability of the selected study area by the high density of OSR crops and the fact that no other attractive flowering crop was available to provide bee forage during the flowering of OSR. The high density of OSR crops and the long agricultural history can be seen as a realistic worst case scenario providing the highest possible exposure to OSR found under field conditions which was also verified by the comparison with other OSR cultivation sites in Europe. Due to the representativeness of the study area for other OSR cultivation regions, the findings of the monitoring project are not restricted to the study area but can also be transferred to other OSR cultivation sites in Europe.

## Electronic supplementary material

Supplementary Information
