# Supplementary material for: Large-scale monitoring of effects of clothianidin dressed oilseed rape seeds on pollinating insects in Northern Germany: implementation of the monitoring project and its representativeness
Source: Ecotoxicology. 2016 Sep 27;25(9):1630–47. doi: 10.1007/s10646-016-1724-9 (PMC5093193; doi:10.1007/s10646-016-1724-9)
Supplement: Supplementary file 1 — Supplementary Information [file 10646_2016_1724_MOESM1_ESM.pdf]

Electronic Supplementary Material

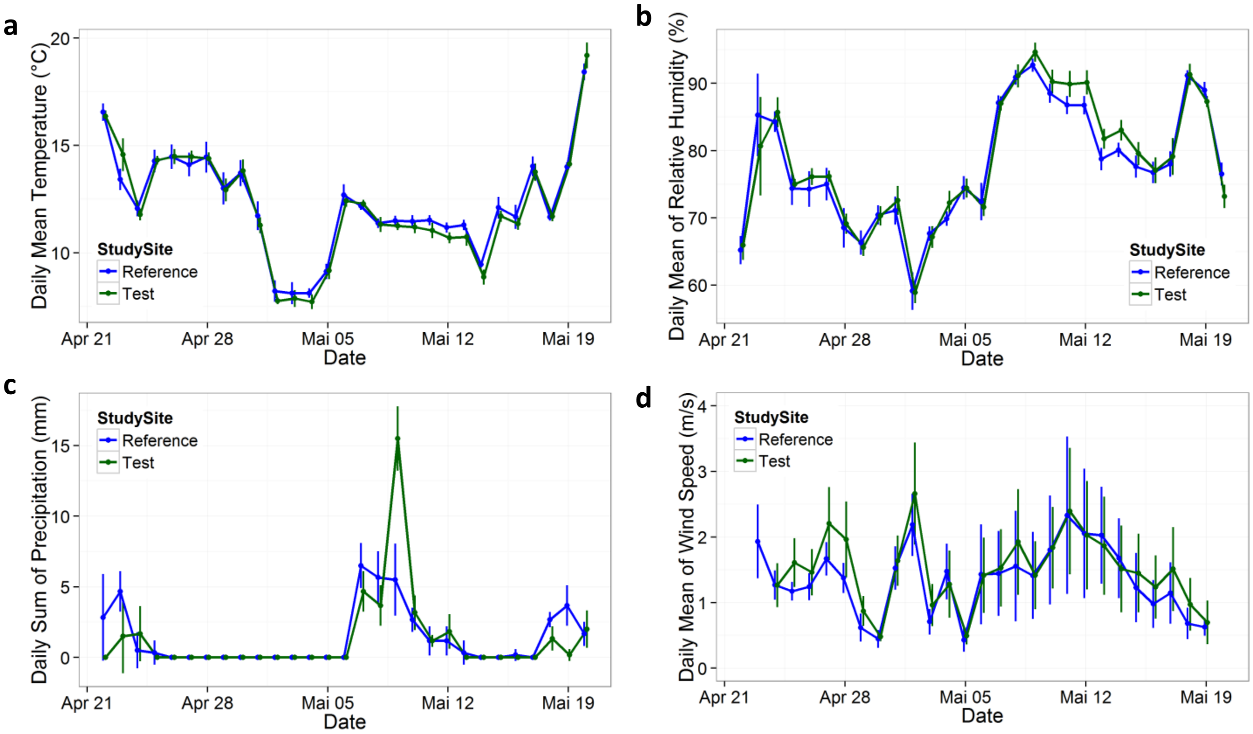

Figure S 1 Weather conditions at the honey bee study locations during the exposure phase. Daily means of temperature (a), relative humidity (b), and wind speed (d), and the daily sum of precipitation (c) averaged per study site. Error bars indicate the 95 % confidence intervals per study site and day and are slightly offset to avoid overlapping.

Mean air temperature (°C)

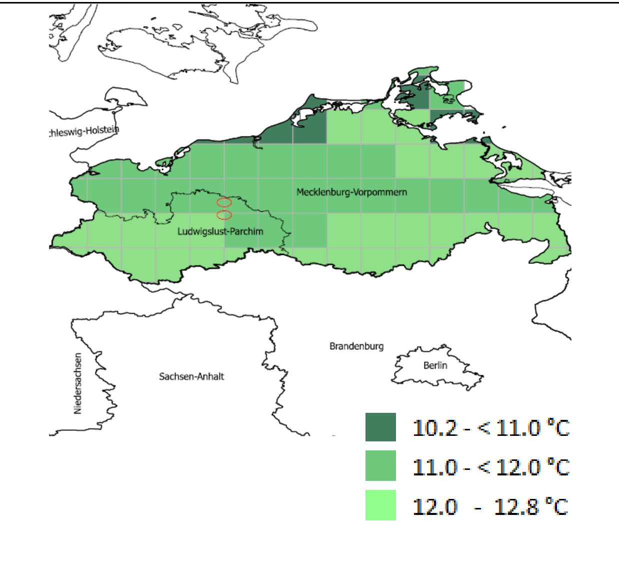

Maximum air temperature (°C)

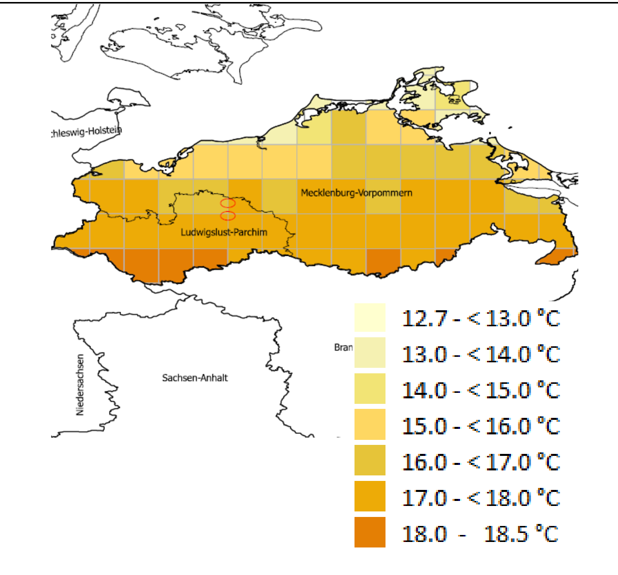

Minimum air temperature (°C)

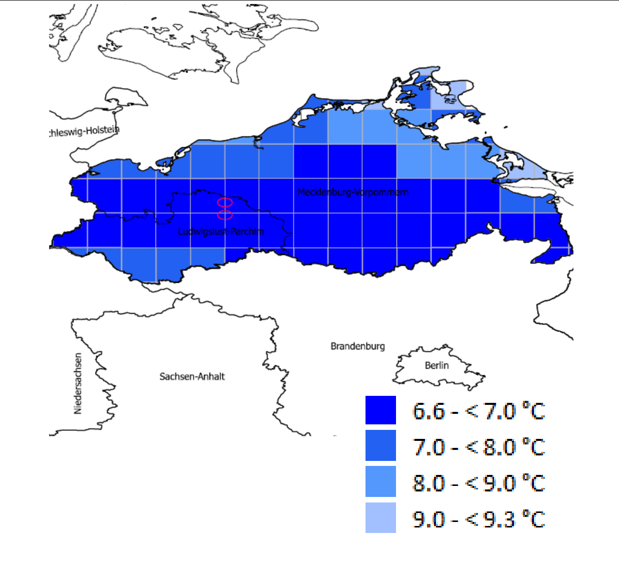

Figure S 2 Long-term average (2000-2013) mean, maximum, and minimum air temperature (°C) during the 30 days flowering period of winter oilseed rape in the study area (red circles), the district of Ludwigslust-Parchim and the federal state Mecklenburg-West Pomerania.

Precipitation

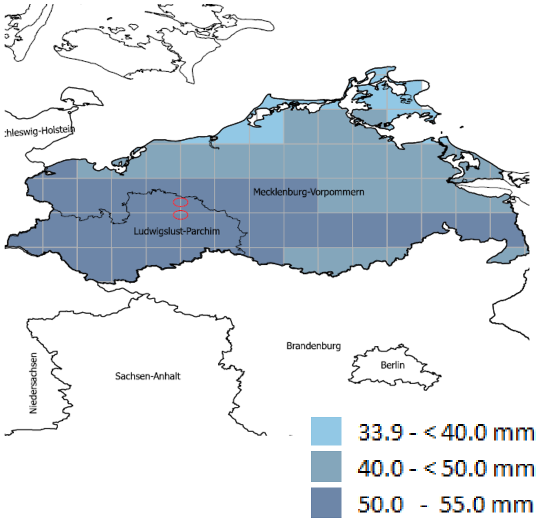

Solar radiation

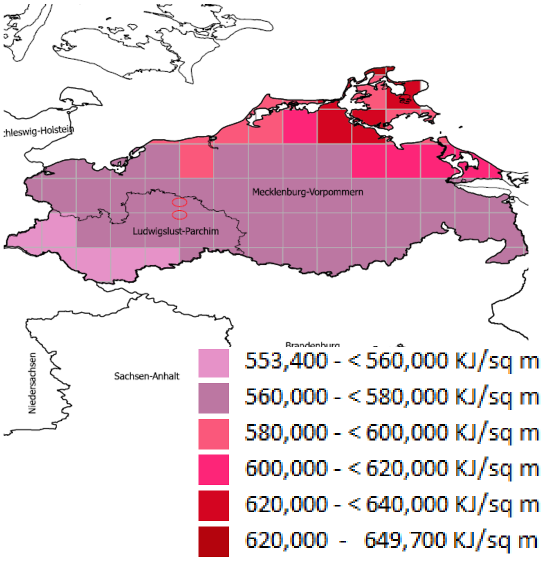

Figure S 3 Long-term average (2000-2013) precipitation (mm) and solar radiation (KJ/m^2) during the 30 days flowering period of winter oilseed rape in the study area (red circles), the district of Ludwigslust-Parchim and the federal state Mecklenburg-West Pomerania.

Table S 1 Characterisation of OSR seeds. The flowering time is coded as follows *2*: early, *3*: intermediate, *4*: late. For study fields outside the core area some information was not available and is indicated by *a*. *b* indicates that the clothianidin loading of seeds was not analysed. *c* The varieties at the demonstration field T13 which were also drilled to the study field T10, are not allowed to carry a batch number. *d* Note that the varieties *Anisse* and *ES Alegrie* of study field T13 were not dressed with clothianidin but the dressing contained thiamethoxam. The value indicated refers in these two cases to the respective loading with thiamethoxam in mg/kg seeds.

Study Site	Study Field	OSR Variety	Seed manufacturer	Batch number	Sowing Date	Flowering Time	Plot Size (ha)	TSW (g)	Amount Sowed (kg)	Seed Density (kg/ha)	Clothianidin Loading (mg/kg seed)
Reference	R1	Sherpa	Rapool	DE 013-1471447	21.08.2013	3	16.9	8.5	64.9	3.8	113
	R2	Sherpa	Rapool	DE 013-1471447	19.08.2013	3	26.7	8.5	99.2	3.7	126
	R3	Genie	Rapool	DE 053-323088	13.08.2013	3	34.0	7	102.3	3.0	19
	R4	Genie	Rapool	DE 053-323088	14.08.2013	3	33.5	7	90.4	2.7	24
	R5	Sherpa	Rapool	DE 013-1471447	19.08.2013	3	21.8	8.5	83.9	3.9	190
	R6	Avatar	Rapool	DE 013-1471584	15.08.2013	2	10.8	6.8	34.4	3.2	226
	R7	Andromeda	LG	DE 063-1723110	21.08.2013	2	97.3	5.5	167.3	1.7	24
	R8	Genie	Rapool	DE 053-323088	17.08.2013	3	10.3	7	27.9	2.7	13
	R9	Sherpa	Rapool	DE 013-1471447	22.08.2013	3	36.9	8.5	153.6	4.2	97
	R10	Genie	Rapool	DE 053-323088	18.08.2013	3	67.7	7	168.6	2.5	15
	R11	Compass	Rapool	DE 053-323022	19.08.2013	3	37.7	6.7	97.1	2.6	23
	R12	Compass	Rapool	DE 053-323022	19.08.2013	3	8.96	6.7	24.3	2.7	19
	R13	Compass	Rapool	DE 053-323022	20.08.2013	3	32.8	6.7	81.0	2.5	19
	R14	PR 46 W20	Pioneer	DE 033-4620100W	19.08.2013	4	69.6	4.1	112. 9	1.6	1
	R15	PR 46 W20	Pioneer	DE 033-4620100W	19.08.2013	4	15.5	4.1	26.1	1.7	1
	R16	Genie	Rapool	DE 053-323088	16.08.2013	3	65.3	7	162.4	2.5	14
	R17	Genie	Rapool	DE 053-323088	16.08.2013	3	37.6	7	93.4	2.5	27
	R18	Sherpa	Rapool	a	20.08.2013	3	12.9	a	a	a	b
	R19	Sherpa	Rapool	a	20.08.2013	3	4.8	a	a	a	b

Study Site	Study Field	OSR Variety	Seed manufacturer	Batch number	Sowing Date	Flowering Time	Plot Size (ha)	TSW (g)	Amount Sowed (kg)	Seed Density (kg/ha)	Clothianidin Loading (mg/kg seed)
Test	T1	Compass	Rapool	DE 053-323003	29.08.2013	3	38.0	6.1	114.4	3.0	6515
		Sherpa	Rapool	DE 053-323307	29.08.2013	3	11.8	5.6	31.9	2.7	4387
	T2	Xenon	Rapool	DE 013-1471531	15.08.2013	2	46.8	5.3	103.6	2.2	8144
	T3	Xenon	Rapool	DE 013-1471531	14.08.2013	2	198	5.3	366.6	1.9	8215
	T4	PR 46 W20	Pioneer	DE 033-4620008W	21.08.2013	4	15.5	6.6	39.6	2.6	8808
	T5	PR 46 W20	Pioneer	DE 033-4620008W	20.08.2013	4	10.5	6.6	26.4	2.5	7651
	T6	PR 46 W20	Pioneer	DE 033-4620008W	20.08.2013	4	25.7	6.6	66.0	2.5	8308
	T7	Sherpa	Rapool	DE 013-1471502	13.08.2013	3	64.8	6.3	195.7	3.0	7448
	T8	PR 46 W20	Pioneer	DE 032-4621999W	13.08.2013	4	61.5	4.4	124.3	2.0	6980
	T9	Arsenal	LG	DE 063-1723151	24.08.2013	2	12.0	8.37	55.2	4.6	8071
		PR 46 W20 Batch 1	Pioneer	DE 032-462182W	24.08.2013	4	12.1	4.7	31.5	2.6	8699
		PR 46 W20 Batch 2	Pioneer	DE 032-4622005W	24.08.2013	4	5.0	4.9	12.5	2.5	8369
	T10	Mercedes	Rapool	DE 013-1471872	25.08.2013	4	4.5	5.4	22.5	5.0	5142
		Alister	Syngenta	c	25.08.2013	3	1.0	5.23	5	5.0	8648
		Saveo	Syngenta	c	25.08.2013	3	3.8	4.57	10	2.6	8855
		Vesuvio	Syngenta	c	25.08.2013	3	3.8	4.57	10	2.6	7940
		Mix 1	-	c	25.08.2013	3	25.9	6.26	44.9	1.7	8000
	T11	Sherpa	Rapool	DE 013-1471500	22.08.2013	3	42.6	6.1	123.2	2.9	8107
	T12	Arabella	LG	DE 063-1723139	20.08.2013	2	12.5	8.34	70	5.6	7238

Study Site	Study Field	OSR Variety	Seed manufacturer	Batch number	Sowing Date	Flowering Time	Plot Size (ha)	TSW (g)	Amount Sowed (kg)	Seed Density (kg/ha)	Clothianidin Loading (mg/kg seed)
Test	T13	Marquis	SW	c	16.08.2013	3	0.75	5.5	4	5.3	7236
		Mascara	SW	c	16.08.2013	4	0.78	5.9	4	5.2	8539
		PX 104	Pioneer	c	16.08.2013	3	0.80	6.3	4	5.1	7111
		Arabella	LG	c	16.08.2013	2	0.79	5.97	4	5.1	8927
		PX109	Pioneer	c	16.08.2013	3	0.80	7	4	5.0	8554
		Arsenal	LG	c	16.08.2013	2	0.81	7.45	4	4.9	7533
		Anisse	Euralis	c	16.08.2013	2	0.82	7.7	4	4.9	2958[d]
		Trinity	SW	c	16.08.2013	3	0.83	5.3	3	3.6	10824
		Patron	Bayer	c	16.08.2013	3	0.84	4.5	3	3.6	8007
		Sherlock	KWS	c	16.08.2013	2	0.85	4.2	3	3.5	8880
		Raptor	KWS	c	16.08.2013	2	0.87	4.05	3	3.5	7855
		ES Alegrie	Euralis	c	16.08.2013	2	0.88	4.5	3	3.4	2959[d]
		Letitia	Bayer	c	16.08.2013	3	0.89	4.5	3	3.4	7805
		Marathon	Rapool	c	16.08.2013	2	0.90	4.85	3	3.3	8611
		Genie	Rapool	c	16.08.2013	3	0.91	4.6	3	3.3	6561
		Mercedes	Rapool	c	16.08.2013	4	0.92	5.4	3.7	4.0	7767
		Comfort	Rapool	c	16.08.2013	4	0.93	5.93	3.7	4.0	8654
		Midas	Rapool	c	16.08.2013	2	0.94	6.1	3.7	3.9	9486
		Sherpa	Rapool	c	16.08.2013	3	0.95	5.7	3.7	3.9	10138
		Foxx	Rapool	c	16.08.2013	3	0.96	6	3.7	3.8	9492
		Avatar	Rapool	c	16.08.2013	2	0.97	6.8	4.9	5.0	8914
		Visby	Rapool	c	16.08.2013	2	0.98	8.8	4.9	5.0	8959
		Mix 2	-	c	16.08.2013	3	14.4	5.37	43.5	3.0	6800

Study Site	Study Field	OSR Variety	Seed manufacturer	Batch number	Sowing Date	Flowering Time	Plot Size (ha)	TSW (g)	Amount Sowed (kg)	Seed Density (kg/ha)	Clothianidin Loading (mg/kg seed)
**Test**	T14	Marathon (Elado+DMM)	Rapool	-	17.08.2013	2	12.0	4.85	25.8	2.2	7844
		Marathon (Elado+TMTD)	Rapool	DE 053-323193	17.08.2013	2	12.5	7.2	28.0	2.2	7871
	T15	Visby Batch 1	Rapool	DE 053-323326	22.08.2013	2	45.0	8.3	218.6	4.8	6663
		Visby Batch 2	Rapool	DE 053-323544							6265
		Sherpa	Rapool	DE 012-147030		3	1.50	5.7	7.2		9082
	T16	Adriana	LG	DE 033-0050003	16.08.2013	3	15.0	6.1	33.4	2.2	7581
	T17	Avatar	Rapool	DE 053-323450	19.08.2013	2	55.4	6.2	178	3.2	8148
	T18	Sherpa	Rapool	a	22.08.2013	3	37.8	a	a	a	b
	T19	Visby	Rapool	a	22.08.2013	2	67.6	a	a	a	b
	T20	Sherpa	Rapool	a	24.08.2013	3	35.6	a	a	a	b
	T21	Dimension	Rapool	a	24.08.2013	2	33.3	a	a	a	b
	T22	Visby	Rapool	a	15.08.2013	2	96.7	a	a	a	b
	T23	Arabella	LG	a	20.08.2013	2	16.1	a	a	a	b
	T24	Adriana	LG	a	19.08.2013	3	45.3	a	a	a	b
	T25	Compass	Rapool	a	24.08.2013	3	4.47	a	a	a	b
	T26	Sherpa	Rapool	a	28.08.2013	3	9.56	a	a	a	b
	T27	Sherpa	Rapool	a	28.08.2013	3	9.13	a	a	a	b
